# Cocaine as a Local Anæsthetic in the Operation for Cleft Palate

**Published:** 1885-03

**Authors:** Roswell Park

**Affiliations:** Professor of Surgery in the Medical Department, University of Buffalo


### COCAINE AS A LOCAL ANÆSTHETIC IN THE OPERATION FOR CLEFT PALATE.

BY ROSWELL PARK, A. M., M. D.

*Professor of Surgery in the Medical Department, University of Buffalo.*

While I am well aware that there is no longer extreme novelty attached to the use of cocaine, I have yet to learn that it has been used as a local anæsthetic in an operation so trying and prolonged as that for the relief of extensive fissure of the hard and soft palate, and consequently think it worth while to report the following case:

J. H., age 22, of Medina, N. Y., was sent to me with a view to the relief of the condition of the roof of his mouth. I found a very typical but not remarkably aggravated fissure, involving the entire soft palate and the posterior half of the hard, the cleft being in the exact middle line, so that the uvula was evenly divided. There was a certain amount of naso-pharyngeal catarrh, with some chronic enlargement of the tonsils, but nothing to contra-indicate operation, toward which all indications pointed favorably. The young man was afflicted with a distressing stammer in his speech, making at times a grunting sound almost painful to listen to. Added to his hesitating speech, the peculiar nasal tone, due to his oral defect, caused him to be seldom or never heard with pleasure.

Jan. 17th, 1885, in my surgical clinic at the Buffalo General Hospital, I operated before my class. At precisely 11 A. M. the first application was made of a four per cent. fresh solution of a freshly procured specimen of Merck's manufacture. This was painted over the parts with a camel's hair pencil. The application was repeated, at ten-minute intervals, till four had been made. I began to operate at 11.35. First of all, with a hypodermic syringe, I injected about four minims of the same solution in a portion of the soft palate, on either side, in a location which I knew I should not have to interfere with during operation. The ordinary and recognized procedures were then gone through with, with only such delays as were required for freeing the mouth and throat from blood, or for resting his mouth for a short time from the constraint imposed by the mouth gag. Twice during the procedure was the solution brushed on, after wiping away blood and mucus.

Paring of the edges of the fissure, the lateral incisions necessary for uranoplasty, freeing the periosteum from the bony palate, tenotomy of the tensores palati, section of the muscles composing the faucial pillars, and introduction of sutures, were all performed with such deliberation as would ensure thorough work and permit demonstration to as many as could see the steps of the operation. Four silver shotted sutures and three of silk were introduced, by means of which the fissure was perfectly closed, and without undue tension. The entire operation, including "waits," occupied about an hour.

I will not say that the patient felt *no* pain, for when the soft parts were loosened from the hard palate, and when the faucial pillars were cut he certainly squirmed in an excusable way, nor can I institute any relative test as to the exact amount of his suffering, since he had never before been subjected to any operation; but I am convinced that acute sensibility was greatly obtunded, and the whole proceeding made more expeditious and less painful than it would otherwise have been. Nothing but a general anæsthetic could obviate the painful annoyance of having the jaws so widely separated for the greater part of an hour, and this discomfort could not be spared him. At the close of the sitting he was rather faint, but a part, at least, of his faintness might well be ascribed to the amount of cocaine introduced through the hypodermic needle.

Altogether, the experiment, from a physiological view alone, was not without interest, while clinically it was quite gratifying.

I may add that the result was perfect union of the whole line of sutured edges, the stitches being removed on the fifth and sixth days. Anatomically the result is therefore perfect; but I have little hope here, or in any similar case, of remedying the peculiarly disagreeable nasal tone of the speaking or singing voice.

